# An Enlarged Parietal Foramen in the Late Archaic Xujiayao 11 Neurocranium from Northern China, and Rare Anomalies among Pleistocene *Homo*

**DOI:** 10.1371/journal.pone.0059587

**Published:** 2013-03-18

**Authors:** Xiu-Jie Wu, Song Xing, Erik Trinkaus

**Affiliations:** 1 Key Laboratory of Evolutionary Systematics of Vertebrates, Institute of Vertebrate Paleontology and Paleoanthropology, Chinese Academy of Sciences, Beijing, China; 2 Department of Anthropology, Washington University, Saint Louis, Missouri, United States of America; Museo Nazionale Preistorico Etnografico ‘L. Pigorini’, Italy

## Abstract

We report here a neurocranial abnormality previously undescribed in Pleistocene human fossils, an enlarged parietal foramen (EPF) in the early Late Pleistocene Xujiayao 11 parietal bones from the Xujiayao (Houjiayao) site, northern China. Xujiayao 11 is a pair of partial posteromedial parietal bones from an adult. It exhibits thick cranial vault bones, arachnoid granulations, a deviated posterior sagittal suture, and a unilateral (right) parietal lacuna with a posteriorly-directed and enlarged endocranial vascular sulcus. Differential diagnosis indicates that the perforation is a congenital defect, an enlarged parietal foramen, commonly associated with cerebral venous and cranial vault anomalies. It was not lethal given the individual’s age-at-death, but it may have been associated with secondary neurological deficiencies. The fossil constitutes the oldest evidence in human evolution of this very rare condition (a single enlarged parietal foramen). In combination with developmental and degenerative abnormalities in other Pleistocene human remains, it suggests demographic and survival patterns among Pleistocene *Homo* that led to an elevated frequency of conditions unknown or rare among recent humans.

## Introduction

As a result of the description and diagnosis of developmental and degenerative abnormalities in Pleistocene human remains, it has become evident that skeletal and dental reflections of the stresses of a Pleistocene foraging existence are ubiquitous among these remains. These lesions include principally non-specific developmental growth arrest indicators (dental enamel hypoplasias and transverse lines), trauma (minor and pronounced), osteoarthritis (use-related and posttraumatic), and dentoalveolar lesions (periodontal degenerations and carious lesions). Yet, there has emerged a growing sample of abnormalities, not all strictly pathological (in the sense of affecting function), that appear collectively to be unusually common among these Pleistocene humans, given the fragmentary nature of the human fossil record and the dearth of specimens.

In this context, we describe and diagnose a neurocranial variant in the early Late Pleistocene Xujiayao 11 partial cranium from northern China, an enlarged parietal foramen connecting with a wide vascular sulcus. Although enlarged parietal foramina are known, if rare, among recent humans [1], they have not been previously reported among Pleistocene humans.

## Materials and Methods

### The Preservation and Identification of Xujiayao 11

**Figure 1:**
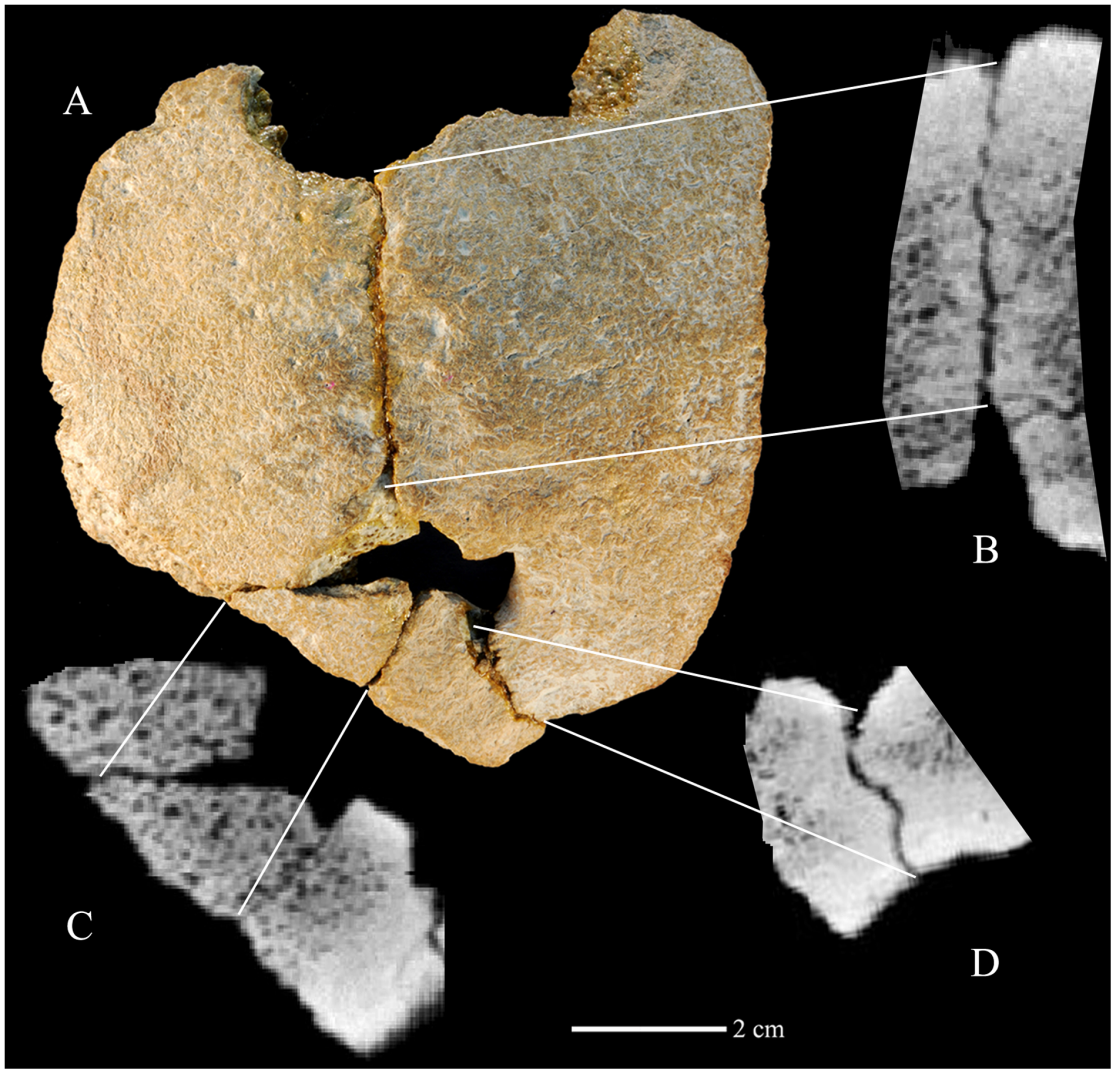
The Xujiayao 11 parietal bones. Exterior view (A). CT horizontal section images showing the linear sagittal suture (B), the posterior left postmortem breaks (C), and the posterior right oblique sagittal suture (D). Anterior is above.

Xujiayao 11 (IVPP PA-1494) is a highly mineralized human neurocranial fossil [2] that was found in five pieces (Figure 1). The two larger pieces join tightly along a suture, and the larger of the remaining pieces similarly joins along a suture to the largest element. The fourth piece connects across postmortem breaks, and the last one joins to the endocranial edge of the second largest piece. Although there was minor bone loss across the separated sutures and breaks, especially endocranially, the amount was minimal; there is no resultant distortion.

**Figure 2:**
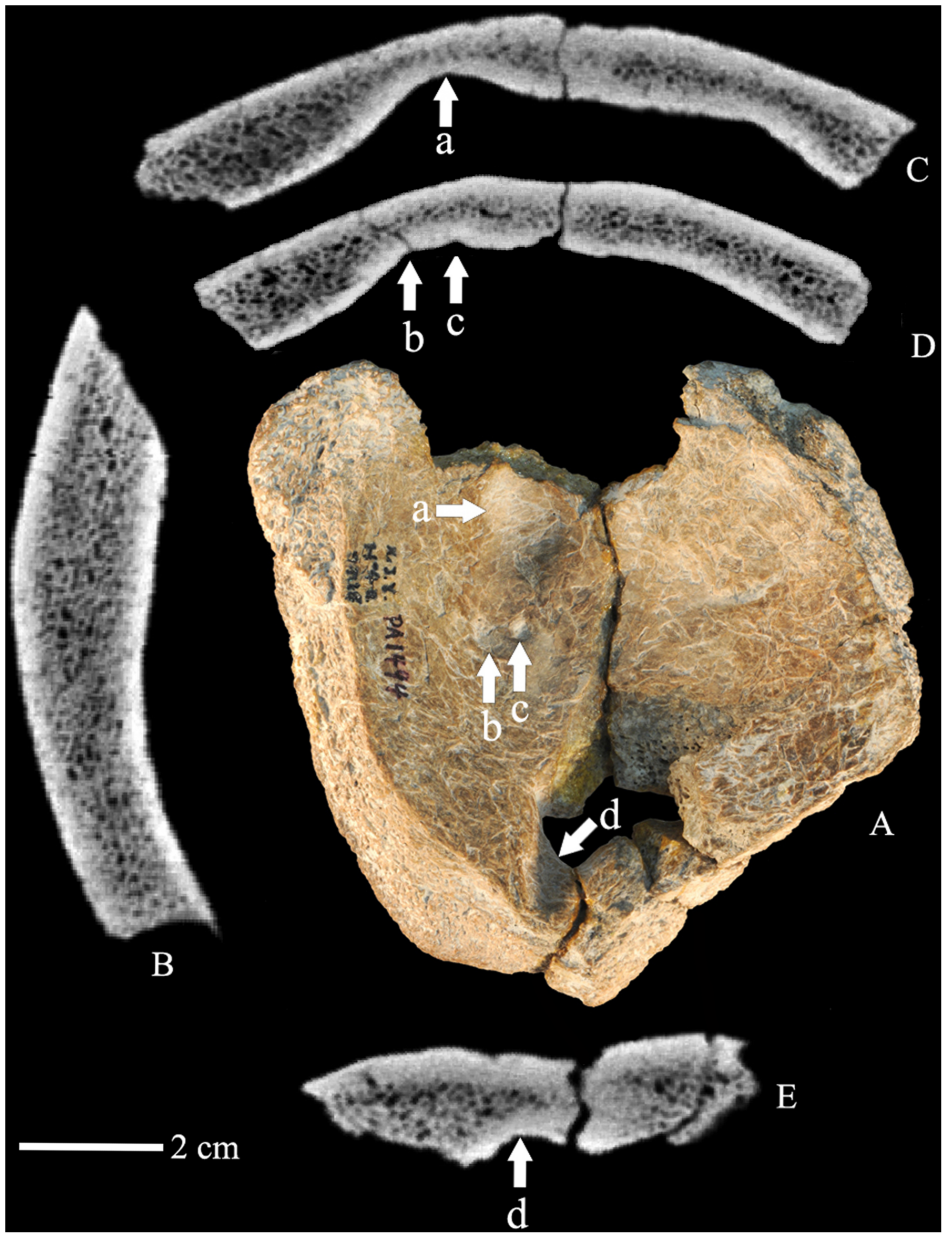
The Xujiayao 11 parietal bones. Interior view (A). CT sagittal section image showing the thickness of the bone (B). CT coronal section images (C, D, E) showing the large Pacchionion depression (a), the two small granular foveolae (b, c) and the wide venous sulcus (d) in the bone. Anterior is above.

The assembled pieces of Xujiayao 11 therefore represent a pair of neurocranial vault bones, joined along a 41 mm long, linear and partially obliterated suture. The specimen extends 40–50 mm to either side of the suture; the maximum preserved length is 91 mm on one side and 74 mm on the other side (Figure 1). There is no evidence of meningeal sulci endocranially, but there is a large, ∼25 mm by ∼16 mm, Pacchionian depression along the suture on the largest of the pieces (Figure 2a). Adjacent to the Pacchionian depression are two depressions (Figure 2b and 2c), each identified as a granular foveola from arachnoid granulations. The assembled piece is therefore identified as the posteromedial right and left parietal bones with a posterior section of the sagittal suture, extending from the middle of the bregma-lambda arc to the region above lambda.

There is no evidence of the lambdoid suture on the preserved posterior portions of the parietal bones. Moreover, the sagittal suture does not continue posteriorly in a straight line, but it ends at a lacuna in the bone. There is then an oblique section of a suture to the posterior right (Figure 1). It is 15.3 mm in length and at a ∼25° angle with the more anterior and linear sagittal suture. Given the absence of the lambdoid suture at the same anteroposterior position on the left side, it is inferred that this short sutural segment represents a right deviation of the posterior sagittal suture, anterior of lambda. The absence of the lambdoid suture prevents assessment of how this sutural deviation may have been related to other sutural configurations in the cranium.

The parietal bone appears very thick, ranging in thickness from ∼9.5 mm near the sagittal suture to a maximum of 14.6 mm laterally. At the thickest point, the Xujiayao 11 external table is 3.3 mm thick, the internal table is 2.4 mm thick, and hence the diploë is 8.9 mm thick (see CT slices in Figure 2). Close to the parietal eminence, it is 9.6 mm thick, with internal and external table thicknesses of 2.9 and 2.6 mm and a diploic thickness of 4.1 mm. These table to diploë proportions indicate an adult, possibly an older individual [3].

### The Context of Xujiayao 11

The Xujiayao 11 neurocranial specimen was excavated during the 1977 field season [2] at the Xujiayao site (Locality 74093 in the village of Houjiayao; 40°06′02″ N, 113°58′39″E). The site is situated on the west bank of the Liyi River, a small tributary of the Sanggan River, near the northwestern boundary of the Nihewan Basin, northern China. The sequence of deposits consists of open-air fluviatile and lacustrine deposits, with erosional surfaces present within stratigraphic layers of sandy or silty clay.

During excavations in 1976, 1977 and 1979, thousands of lithic artifacts, abundant faunal remains, and 19 fragmentary human remains were unearthed from a sloping layer of yellowish-green clay, that is between 8 and 12 m below the modern surface [4], [5]. The assemblage contains a diverse colder climate vertebrate faunal assemblage that is dominated by late Middle and Late Pleistocene species [5], and the climatic inference is supported by palynological remains [6]. However, more temperate fauna, such as *Cervus nippon*, are also evident in the assemblage.

Taphonomic analysis of the equid and artiodactyl remains [7] has shown that the frequencies and distributions of cut-marks, tooth-marks, percussion-marks and bone fragmentation fall within the expected ranges for assemblages generated principally by humans with only secondary carnivore involvement. There is little evidence of fluviatile transport (1.5% and 2.5% of the equid and artiodactyl bones), but there are more common indications of trampling and/or sedimentary abrasion (22.8% and 23.2% respectively). Nine of the human remains show little or no evidence of weathering or surface erosion, but the other ten (including Xujiayao 11) have had their surfaces slightly altered with varying amounts of surface weathering, root etching and/or edge abrasion. In none of them do the surface alterations obscure the morphological features or erode through the surface cortical bone.

A precise radiometric age for the archeological level has remained elusive. Uranium-series dating on *Equus* sp. and *Coelodonta antiquitatis* tooth enamel provided mean ages between ∼104 ka BP and ∼125 ka BP [8], within Marine Isotope Stage (MIS) 5. The deposits are above a paleomagnetically reversed sequence, below ∼15 mm, which has been interpreted as the early MIS 5 Blake Excursion [9], 119–126 ka BP [10]. More recently preliminary optically stimulated luminescence (OSL) dating of the archeological horizon provided late MIS 4 ages (60±8 and 69±8 ka BP) [11]. The Xujiayao human remains therefore likely derive from early Late Pleistocene (MIS 5 to 4) deposits. Morphologically, they represent late archaic humans and are distinct from *H. erectus* and early modern humans [2], [5], [12], [13].

## Methods

The Xujiayao 11 human fossil, in the Institute of Vertebrate Paleontology and Paleoanthropology (IVPP), Chinese Academy of Sciences (specimen PA-1494), was analyzed using a digital microscope (KH-8700; Hirox, Tokyo), computerized tomography (CT), and scanning electron microscopy (SEM). It was CT scanned in coronal orientation by use of a high-resolution industrial CT scanner (450 kV-ICT; made by the Institute of High Energy Physics, Chinese Academy of Sciences) at the IVPP. The CT scan parameters were: X-ray tube voltage: 400 kV; X-ray tube current: 1.5 mA; slice thickness 0.3 mm. Three hundred and forty seven slices were obtained. The primary scanned slice data were processed with 2D reconstruction software made by the Institute of High Energy Physics, Chinese Academy of Sciences. The pixel matrix of the complete set of slices is 2048×2048, and the color depth is 8 bits. The reconstruction diameter of each slice is 409.6 mm and each pixel size is 0.2×0.2 mm. On a Dell Graphics Workstation, the 3D reconstructions were created by post processing the CT data and running Mimics 15.1 (Materialise NV, Leuven) to extract the maximum information concerning internal cranial features, osseous distribution and the perforation. The Rapidform software program (INUS Technology, Seoul) was used to modify the 3D models. The SEM images were generated on a S-3700N (Hitachi, Tokyo) scanning electron microscope, with a SE resolution of 10 nm at 3 kV. The working distance was 34.7–41.8 mm, with magnifications of 20x–30x.

## The Xujiayao 11 Parietal Alterations

### The Parietal Lacuna

**Figure 3:**
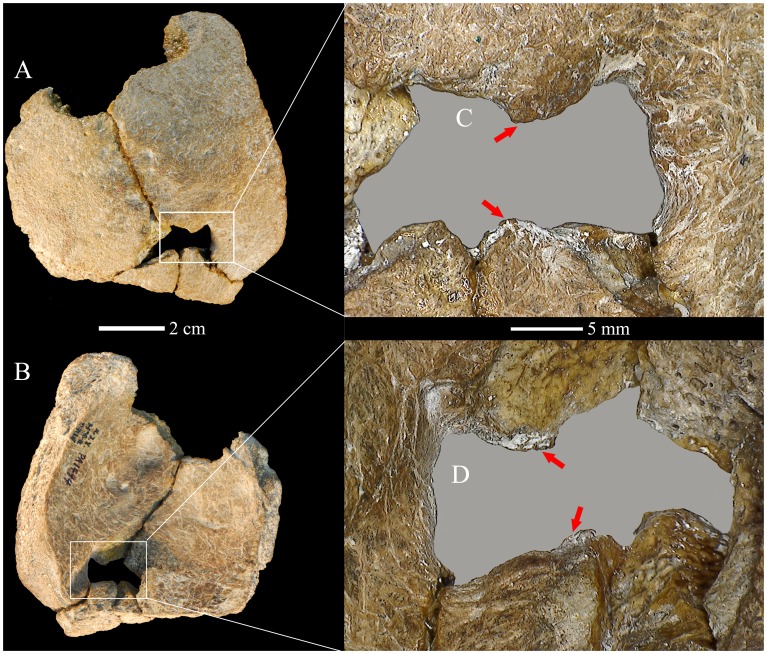
The right parietal perforation of Xujiayao 11. Exocranial (A) and endocranial (B) details of the opening. The bone is oriented with the opening approximately horizontal, such that anterior is above-left in the exocranial view and above-right in the endocranial view. The rounded and beveled edge is evident in the external table (C). The vascular groove is evident on the inner table (D). The arrows delimit the preserved right rounded margins of the hole, to distinguish it from the left postmortem breakage of the margins.

**Figure 4:**
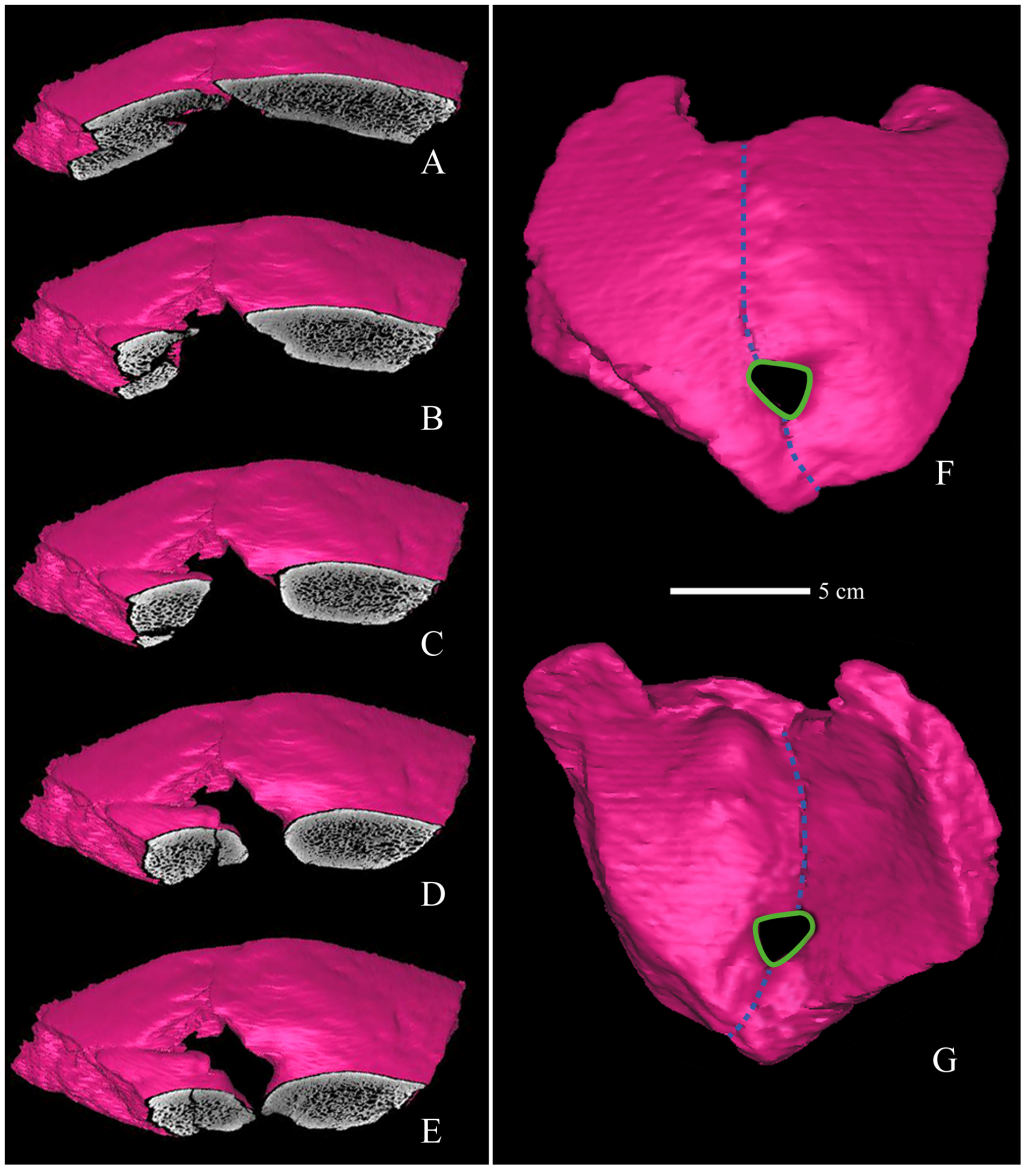
CT reconstruction of the Xujiyao 11 parietal bones with sequential coronal slices through the perforation. The slices extend from the anterior edge of the opening (A) to close to the posterior margin (E). A 3D CT reconstruction of the specimen is shown in external (F) and internal (G) views, with the postmortem breakage filled in and the sagittal suture line provided.

As noted above, there is a lacuna in the posterior parietal bones, in line with the sagittal suture and extending to either side (Figure 3). On the left side, the edges are broken with exposure of diploë, indicating a postmortem break between the larger and smaller of the pieces. On the right side, however, the edges are rounded; the limits of the rounded edges of the lacuna are indicated in Figure 3. There is continuous cortical bone for ∼9 mm around the margin of the hole from the external table to the internal one (Figures 4C and 4D). The exocranial to endocranial cortical bone contrasts with the exposure of the diploë, evident in Figure 4A and on the left side of the hole in Figures 4B and 4C.

**Figure 5:**
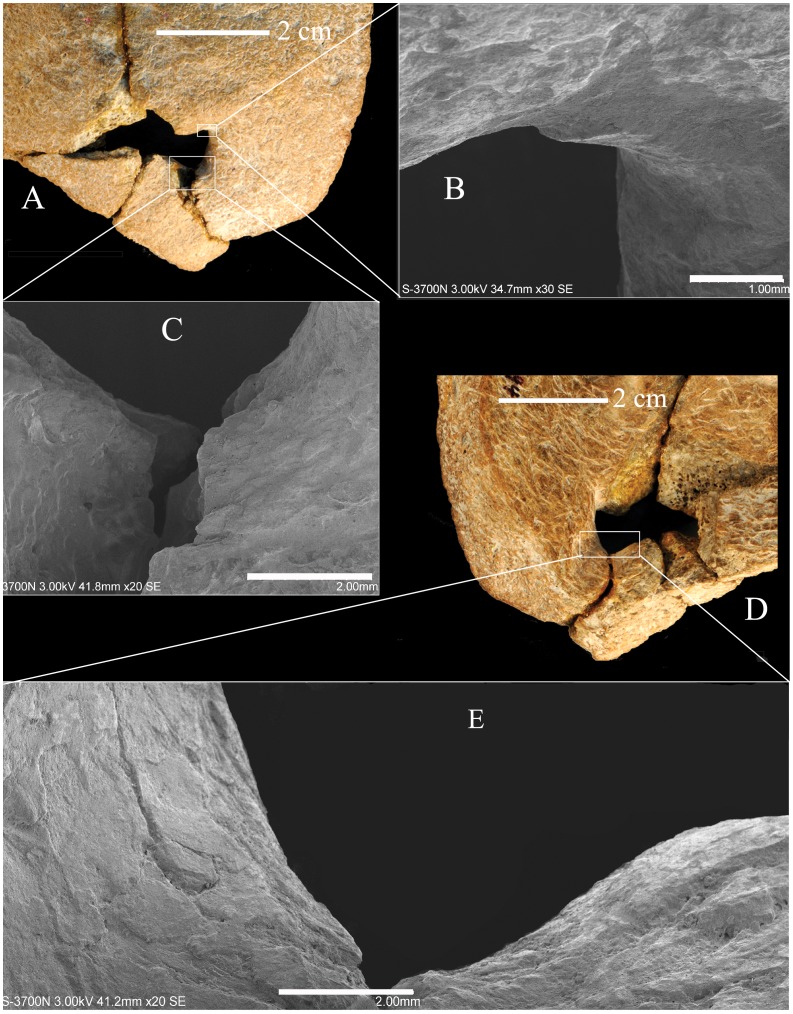
The Xujiayao 11 posteromedial parietal lacuna, with SEM details of the margins. Exocranial (A) and endocranial (D) views of the opening with details of the anterolateral corner (B), the posterolateral sutural margin (C), and the posterolateral endocranial sulcus margin (E). Scale bars are 1 mm for B and 2 mm for C and E.

Given that the bone sustained minor postmortem abrasion of the surfaces, the edges of the rounded portion of the hole were investigated using scanning electron microscopy (Figure 5). There is some minor loss of surface bone around the right margins of the lacuna (Figures 5B and 5E), especially where the short posterior segment of suture meets the edge of the hole (Figure 5C). Yet, it is apparent that there is principally original surface bone preserved and that the original surface contour of the edge rounding is intact.

Therefore, based on the macroscopic rounding, the continuous exocranial to endocranial cortical bone, and the minimal surface bone loss, a portion of the right side of the lacuna is the margin of an antemortem perforation through the parietal bone. The original size of the opening is interpolated in Figures 5F and 5G, using the rounded contours and the left limits of those contours. The sagittal dimensions of the hole are 10.4 mm exocranially and 9.4 mm endocranially. Given the reconstructed margin, the coronal diameters are ∼13.3 mm externally and ∼10.8 mm internally.

### The Posterior Sulcus

Alongside of the posterior right-deviated sagittal suture, endocranially there is a moderately deep sulcus, 12 mm in length and 7–8 mm wide, which extends from the posterior edge of the antemortem lacuna to the posterior right margin of the parietal piece (Figure 2d). The sulcus is deepest at the edge of the lacuna, and it then approaches the endocranial surface at the broken edge of the bone. The transverse CT slice section (Figure 2E) indicates that the inner table follows the groove’s rounded contour and continues along the floor of the sulcus.

Given the usual morphology of the posteromedial parietal bones, the sulcus should be for the superior sagittal sinus, following the right-deviated posterior sagittal suture. However, the more anterior sagittal sulcus along the sagittal suture is wide but not pronounced, and the posterior sulcus forms a distinctly rounded depression that extends from within the perforation to the endocranial surface at the posterior postmortem break (Figures 2 and 3). It is also to the side of the suture rather than spanning across it, in contrast to the usual position of a superior sagittal sinus. It therefore could be for a portion of the superior sagittal sinus, but it is more likely for another vessel between the lacuna and a vascular structure within the neurocranial cavity.

### Differential Diagnosis

The Xujiayao 11 individual therefore exhibits a large antemortem perforation or lacuna through the posterior sagittal suture, ∼10 mm in length and ∼11–13 mm in wide, associated with thick cranial vault bones and a distinct vascular groove extending posteriorly from the perforation.

The Xujiayao 11 parietals are thick, but this aspect is not from a pathological process. Older adults frequently evince expanded diploë and thin tables [3], [14]. Moreover, absolutely thick cranial vault bones are common in later Middle and Late Pleistocene archaic humans (parietal eminence thickness: 8.6±2.2 mm, 5.0–17.0 mm, N = 35) [15], [16], and the Xujiayao 11 value of 9.6 mm is close to the mean value. The Xujiayao 11 elevated thickness is also seen in the other mature Xujiayao parietal bone preserving the parietal eminence (Xujiayao 9∶12.9 mm). The increased thickness in Pleistocene human parietal bones is also at times associated with diploic expansion and relative thinness of the tables [17]–[19], including Xujiayao 9 (diploic thickness: 8.0 mm).

A sagittal perforation similar to the one on the Xujiayao 11 parietals, however, has not been previously noted in human fossils. Factors that might cause holes in the vault include: a sutural ossicle, trauma, a tumor, an eosinophilic granuloma, tuberculosis, syphilis, arachnoid granulations, and an enlarged parietal foramina.

Sagittal ossicles are intrasutural, irregular, isolated bones that occur within the sagittal suture [1]. If the perforation was caused by a lost sagittal ossicle, the edges of the margin should be exo- to endocranially flat and serrated. This is different from the oblique, rounded and thinning edges of the Xujiayao 11 hole.

Localized traumatic injuries to the vault can produce a perforation. However, there is no evidence of antemortem fracture, in the form of a depression, a dislocation of bone tissue, or radiating cracks. A Pleistocene traumatic perforation, given the available lithic and organic technology, is also likely to have impacted the dura mater and associated tissues. In samples of prehistoric intentionally trephined crania, those that employed cross-hatching incisions, through the cranial vault and impacting the dura mater, resulted in low survival rates compared to trephinations that scraped the bone down to the meningeal tissues [20]. Documented cases of serious but healed neurocranial trauma in Pleistocene humans [21]–[24] lack endocranial perforation and produced only modest internal table expansion into the endocranial cavity, in addition to their exocranial alterations.

Tumors, an eosinophilic granuloma or a proliferative disorder of the Langerhans cells, can affect the skeletal system and erode the parietal bone from the inner to the outer table. The parietal bone usually shows swelling and a solitary osteolytic lesion [25]. There is no evidence of the kinds of resorptive (or lytic) processes associated with neoplasms directly impinging on the Xujiayao 11 bone, such as osteoblastomas, meningiomas or hemangiomas of the calvarium [26]–[28]. If intradiploic, they also usually show an expansive appearance through the tables, similar to porotic hyperostosis.

There is no evidence of the kinds of processes from tuberculosis or syphilis infections that can affect cranial vault bones. The former leaves areas of bone destruction. The latter produces destructive lesions (caries sicca) of the diploë and external table, and they frequently produces surficial sequestra [29], [30]. The bone lacks the diploic space expansion towards or through the external table associated with porotic hyperostosis [31].

An arachnoid granulation is a normal structure. Large parietal Pacchionian depressions over 1 cm in diameter very rarely extend to the outer table [32]. The margins of a defect caused by a hemorrhagic granulocyte are clear and produce a mild hardening around it, and the thickness of the outer table is smaller than that of the inner table. This is different from the Xujiayao 11 perforation, whose outer diameter is variably larger than the inner one.

An alternative etiology of this perforation is an enlarged parietal foramen (EPF, or *foramina parietalis permagna*), a rare disorder involving abnormal bone development of the skull resulting in other abnormalities [33]. EPF derive from a malformation of the parietal bones, in which normal symmetrical fetal openings in the parietal bones fail to close during the second half of pregnancy [34], [35]. They can be circular, oblique or irregular in shape and occur near the sagittal suture a few centimeters anterior of the lambda, in the vicinity of the normal parietal foramina [36]. The edges of the EPF are often smoothly beveled at the expense of the outer table with a resultant difference in the internal and external measurements of the foramina [37]. They may be asymptomatic, but they are often associated with cerebral venous anomalies, irregular suture fusion and deviations of the sagittal suture [37], [38].

The Xujiayao 11 single perforation is situated on the posterior sagittal suture, close to the usual location of a normal parietal foramen. The edge margins of the perforation are rounded and beveled from outer table to the inner bone. It appears to have connected with the endocranial venous system, as is indicated by the posterior vascular sulcus. The lacuna is also associated with a right deviation of the sagittal suture. Morphologically, the Xujiayao 11 perforation therefore corresponds to an EPF, in terms of its form, position, and probable endocranial vascular connection. Given the fully adult age-at-death of the individual and the even remodeling of the bone around the perforation, the EPF should not have been associated with the individual’s death.

## Discussion

### Enlarged Parietal Foramina

Recorded instances of enlarged parietal foramina (EPF) are rare, occurring in less than 1 in 25,000 cases among extant humans [33]. Usually, they are bilateral and only exceptionally unilateral [39]–[41]. None has been previously documented among Pleistocene *Homo*.

Normal parietal foramina usually transmit emissary veins connecting the occipital veins to the superior sagittal sinus, as well as an anastomosis between the middle meningeal and occipital arteries [1]. Enlarged parietal foramina are not homologous with normal parietal foramina, since they have been observed in conjunction with normally sized and positioned parietal foramina and have a different developmental basis [35], [42]–[44].

More specifically, EPF are the result of a failure of the normal fetal ossification process of the parietal bones, in which there is delayed ossification during rapid endocranial expansion prenatally [34], [35], although they have also been described as forming postnatally [36]. In some cases EPF are asymptomatic, and as such they have been treated as though they are a discrete trait variant of the human cranium [1], [36], [45]. However, they have been associated with a variety of other developmental abnormalities and symptoms, including cranial bifida, cleft palate, persistently wide fontanelles, scalp defects, headaches and seizures [33], [46]. In addition, they may occur with cortical vascular abnormalities and associated cortical defects and cognitive deficits [35], [43].

Multiple studies have documented familial associations for EPF [42], [43], [47]–[49], leading to inferences of their being inherited in an autosomal dominant fashion [33]. EPF have been associated with Saethre-Chotzen syndrome [50] and deletions in chromosome 11 [43]. They are currently understood to result from mutations of the homeobox genes ALX4 (on chromosome 11) and MSX2 (on chromosome 5) [35], [51], [52].

Enlarged vessels have been observed passing through EPF [33], [40], [48], [53]. The large endocranial sulcus extending posterior from the perforation on Xujiayao 11 therefore implies that such vascular abnormalities were present on this individual, but it cannot be determined whether they would have affected deeper cortical structures. There is no evidence of unusual vascular sulci anterior of the perforation or indications of connections with meningeal sulci on the endocranial parietal bones.

It is difficult to imagine that the Xujiayao 11 individual exhibited the more pronounced deleterious conditions sometimes associated with EPF, given human foraging conditions of the earlier Late Pleistocene. However, it remains open whether it was asymptomatic or if any of the less severe associated defects were present, especially given the incomplete nature of the fossil specimen. If any of these serious conditions accompanied the enlarged parietal foramen of Xujiayao 11, it argues for enhanced survival abilities among these Pleistocene humans with deleterious abnormalities.

Independent of the secondary consequences of this developmental defect on the Xujiayao 11 individual, it is a case of a very rare abnormality among recent humans nonetheless appearing in the small sample of posteromedial parietal bones available for pan-Old World Late Pleistocene archaic humans. There are 22 Late Pleistocene archaic human fossils preserving at least one posteromedial parietal bone (Amud 1, La Chapelle-aux-Saints 1, Devil’s Tower 1, Engis 2, Feldhofer 1, La Ferrassie 1, Krapina 2, 5 & 16, Maba 1, La Quina 5, 13, 18 & 34, Shanidar 1, Spy 1 & 10, Subalyuk 2, Tabun 1, Témara 2, Teshik-Tash 1, Vindija 205), 25 including Xujiayao 5, 9 and 11; only Xujiayao 11 exhibits this parietal lacuna. Given an expected frequency of 1 in 25,000, the probability of finding an EPF in this sample is therefore ∼0.001. The probability would increase slightly if the 12 MIS 5 and 4 early modern humans preserving a posteromedial parietal were added to the comparative sample (Aduma 1/3, Bouri 5/1, Liujiang 1, Qafzeh 3, 6, 9, 10, 11 & 15, Skhul 5 & 9, Tam Pa Ling 1). It would decrease to ∼0.0001 if only the three Xujiayao posteromedial parietal bones are considered to be the appropriate reference sample, and decrease further if one considers that unilateral EPF are less common than bilateral ones.

### Pleistocene Human Abnormalities

Independent of the severity of the congenital defects associated with the EPF of Xujiayao 11, and whether it was asymptomatic, EPF are nonetheless a rare condition in extant humans. It is therefore surprising that one would find a case of it among the modest number of archaic human remains known from the Late Pleistocene. Yet, recent documentation and differential diagnosis of abnormalities among Pleistocene humans have highlighted a number of cases of developmental or degenerative conditions that are rare among recent humans, sometimes exceptionally so, or are clearly abnormal yet cannot be diagnosed. Not included here are a variety of pronounced, but otherwise unexceptional, degenerative conditions on Pleistocene human remains [24].

From the Early Pleistocene these unusual cases include the massive perimortem periostitis of KNM-ER 1808 [54], the amelogenesis imperfecta of Garba 4 [55], and the diffuse craniofacial lesions of Dmanisi D3444/D3900 [56]. In the Middle Pleistocene, such abnormalities include the unilateral lambdoid synostosis of Atapuerca-SH cranium 14 [57], the probable torticollis of Salé 1 [58], the unilateral labyrinthine ossification and parietal enlargement of Singa 1 [59], and the lumbar kyphotic deformity, spondylolisthesis, and Baastrup disease in the Atapuerca-SH pelvis 1 [60]. Among Late Pleistocene Middle Paleolithic humans, there is the persistent bregmatic fontanelle and temporal bone asymmetries of Pech-de-l’Azé 1 [61], the irregular lumbar and sacral synchondroses in Kebara 2 [62], sacral and pelvic asymmetries in Regourdou 1 [63], probable dens evaginatus with bilateral P_3_ granulomata in Zhiren 3 [64], and infantile hydrocephalus in Qafzeh 12 [65].

In the Upper Paleolithic with better preservation, cases are more common. Nazlet Khater 2 has congenitally short femora [66]. Tianyuan 1 has undiagnosed bilateral distal femoral crests with tibial muscular irregularities [67]. Dolní Vstonice 15 exhibits multiple femoral and humeral deformities from a systemic dysplasia [22]. Sunghir 3 presents bilaterally foreshortened femora with pronounced anterior curvature plus associated fragility [68], [69]. Dolní Vstonice 16 developed minor cleft palate [22]. Brno 2 had systemic periostitis and femoral diaphyseal asymmetries [70], [71]. Cro-Magnon 1 exhibits multiple cranial and appendicular lytic lesions [72]. The Rochereil 1 child had macrocrania with a large cranial lacuna and dental dysmorphic lesions [73]. The Mal’ta 1 child exhibits delayed fontanelle closure and dental abnormalities [74]. And a chondrodystrophic dwarf was present at Romito [75]. Less pronounced Upper Paleolithic abnormalities include the bilateral scaphotrapezial laxity of Dolní Vstonice 16 [22], congenital calcaneonavicular coalition in Bausu da Ture 1 [76], and bilateral presence of an acromial bone in Villabruna 1 [77].

Some of these abnormalities would have had little effect on the individuals involved, but others would have been more serious and/or systemic. More importantly here, although most of them can be found occasionally in recent human skeletal and/or clinical samples, none of them is a common condition. If each is considered separately, then the probability of finding each one in our paucity of Pleistocene human remains varies from low to extremely unlikely. If they are considered together, the probabilities would multiply, and the likelihood of finding so many unusual and/or rare conditions would become extraordinarily small.

To the extent that these abnormalities can be considered congenital or cannot be securely diagnosed, these considerations raise questions regarding the population dynamics of Pleistocene humans. To what extent could this pattern reflect small, highly inbred populations, which were also demographically unstable, resulting in both the increased appearance of congenital deleterious conditions and in their subsequent disappearance through local population extinction? Demographic instability appears to have been characteristic of most Pleistocene human populations [78]–[80]. It remains unclear, and probably untestable, to what extent these populations were inbred, but close genetic relationships have been suggested for one Neandertal sample [81] and some Upper Paleolithic burial groups [76], [82], [83].

### Conclusions

The single large lacuna along the posterior sagittal suture with an associated wide endocranial sulcus of the early Late Pleistocene older adult Xujiayao 11 parietal bone is best diagnosed as a unilateral enlarged parietal foramen (EPF) with endocranial vascular involvement. As such, this individual may have had additional abnormalities, but that cannot be confirmed given the incompleteness of the specimen. However, it provides an additional Pleistocene fossil human with a rare developmental or degenerative condition, one which may have impacted the individual beyond having a “hole in the head.” In association with other abnormal Pleistocene humans, it indicates an unusually high incidence of rare conditions among these Pleistocene humans. This elevated incidence, to which Xujiayao contributes, in turn raises questions about the paleopathology and population dynamics of Pleistocene *Homo*.
